# Effect of Segmental Abutting Esophagus-Sparing Technique to Reduce Severe Esophagitis in Limited-Stage Small-Cell Lung Cancer Patients Treated with Concurrent Hypofractionated Thoracic Radiation and Chemotherapy

**DOI:** 10.3390/cancers15051487

**Published:** 2023-02-27

**Authors:** Jianyang Wang, Fei Han, Yuchao Ma, Yufan Yang, Yuqi Wu, Zimin Han, Xuejie Xie, Jianrong Dai, Nan Bi, Luhua Wang

**Affiliations:** 1 Department of Radiation Oncology, National Cancer Center/National Clinical Research Center for Cancer/Cancer Hospital, Chinese Academy of Medical Sciences & Peking Union Medical College, Beijing 100021, China; 2 Department of Radiation Oncology, National Cancer Center/National Clinical Research Center for Cancer/Cancer Hospital & Shenzhen Hospital, Chinese Academy of Medical Sciences and Peking Union Medical College, Shenzhen 518116, China

**Keywords:** small cell lung cancer, limited stage, hypofractionated radiotherapy, esophagus sparing, esophagitis

## Abstract

**Simple Summary:**

The “gold standard” for limited-stage small cell lung cancer (LS-SCLC) is 45 Gy in 1.5 Gy twice-daily fractions (HYPER) thoracic radiotherapy (TRT) scheduled concurrently with platinum-etoposide chemotherapy. However, the HYPER TRT regimen has failed to be universally implemented, mainly due to its inconvenience and notably increased acute esophageal toxicity (Grade ≥3, 19–27%). We successfully innovated the segmental abutting esophagus-sparing (SAES) technique in order to reduce the radiation dose of the esophagus. Based on a cohort derived from a prospective phase III clinical trial, the SAES technique significantly reduced severe acute esophagitis (≥Grade 3: 3.3%) in patients receiving hypofractionated (HYPO) radiotherapy (45 Gy in 3 Gy once-daily fractions) concurrently with chemotherapy. Thus, the SAES technique could help to achieve better tolerance of the HYPO schedule and provide good feasibility for dose escalation, which may translate to better local control and prognosis in the future.

**Abstract:**

The aim of the current study is to evaluate the effect of segmental abutting esophagus-sparing (SAES) radiotherapy on reducing severe acute esophagitis in patients with limited-stage small-cell lung cancer treated with concurrent chemoradiotherapy. Thirty patients were enrolled from the experimental arm (45 Gy in 3 Gy daily fractions in 3 weeks) of an ongoing phase III trial (NCT 02688036). The whole esophagus was divided into the involved esophagus and the abutting esophagus (AE) according to the distance from the edge of the clinical target volume. All dosimetric parameters were significantly reduced for the whole esophagus and AE. The maximal and mean doses of the esophagus (47.4 ± 1.9 Gy and 13.5 ± 5.8 Gy, respectively) and AE (42.9 ± 2.3 Gy and 8.6 ± 3.6 Gy, respectively) in the SAES plan were significantly lower than those (esophagus 48.0 ± 1.9 Gy and 14.7± 6.1 Gy, AE 45.1 ± 2.4 Gy and 9.8 ± 4.2 Gy, respectively) in the non-SAES plan. With a median follow-up of 12.5 months, only one patient (3.3%) developed grade 3 acute esophagitis, and no grade 4–5 events happened. SAES radiotherapy has significant dosimetric advantages, which are successfully translated into clinical benefits and provide good feasibility for dose escalation to improve local control and prognosis in the future.

## 1. Introduction

Small cell lung cancer (SCLC) accounts for around 15% of all lung cancer cases [1]. According to the results of the landmark Intergroup 0096 trial, a hyperfractionated twice-daily (HYPER) thoracic radiotherapy (45 Gy in 3 weeks, 1.5 Gy per fraction, BID) with concurrent platinum-etoposide chemotherapy, followed by prophylactic cranial irradiation (PCI) to patients with a good response, has been considered the standard care for limited stage (LS) SCLC [2]. However, because of the perspective of logistical issues, HYPER thoracic radiotherapy (TRT) is not widely adopted [2,3,4]. As a result, despite the known detrimental effect of prolonged overall treatment time with TRT [5,6], the majority of patients still receive conventional fractionated (CF) TRT in 5–6 weeks with a dose range of 50.4–66 Gy in 1.8–2 Gy once-daily fractions (QD) [7,8].

With advanced radiotherapy (RT) techniques such as intensity-modulated radiotherapy (IMRT), a schedule of hypofractionated TRT (HYPO) with shorter treatment time is a commonly used alternative and recommended by the latest National Comprehensive Cancer Network (NCCN) Guidelines (Version 2.2022) [9,10,11,12,13]. As opposed to non-small cell lung cancer (NSCLC), SCLC patients often have bulky mediastinal disease with extensive involvement of mediastinal lymph nodes. Therefore, although IMRT allows optimal normal tissue sparing to mitigate the risk of toxicity, severe acute esophagitis is still the most important dose-limiting toxicity for the radiotherapy of LS-SCLC. Previous studies reported 19–27% of patients undergoing concurrent accelerated chemoradiotherapy (CRT) developed severe acute esophagitis (≥Grade 3) [2,3,4]. With the HYPO TRT, the rate of G3+ acute esophagitis was still high, ranging from 13% to 31% in prospective trials [9,13,14].

Based on these results, we conducted a phase III trial (NCT 02688036) using 45 Gy in 15 fractions QD, which is close to 42 Gy in 15 fractions QD implied in two prospective phase II trials [9,13]. Despite the relatively lower median OS (18–21 months) compared with those of the control group (45 Gy BID) (*p* = 0.61) and INT 0096 trial, the tolerance was acceptable with a follow-up of 36–81 months [9,13]. However, concern is expressed about the risk of late esophageal toxicity for the HYPO schedule, which is associated with poor quality of life in surviving patients. Previous studies reported the use of the contralateral esophagus-sparing technique in CF radiotherapy to reduce the rate of esophagitis [15,16]. However, the whole contralateral esophagus was treated as one integrated organ at risk (OAR), which increases the difficulty of the plan. In the present study, we applied an IMRT-based technique to segmentally spare the abutting esophageal wall from the gross tumour, aiming to evaluate the effect on reducing acute esophageal toxicity in patients with LS-SCLC treated with HYPO TRT (45 Gy in 15 fractions QD) and concurrent chemotherapy.

## 2. Materials and Methods

### 2.1. Patient Eligibility

The study population was derived from the experimental arm of an ongoing, open-label, phase III prospective randomised clinical trial (NCT 02688036), which enrolled patients with histologically confirmed SCLC and clinically staged as LS or I-IIIB according to the AJCC cancer staging 7th edition before treatment [17]. This trial was designed to determine whether HYPO TRT (45 Gy in 3 Gy QD, experimental arm) has the same efficacy as CF TRT (60 Gy in 2 Gy QD, controlled arm) in patients with LS-SCLC. Detailed information on the inclusion and exclusion criteria is listed in Supplementary Table S1. Patients with a clinical target volume (CTV) ≤1 cm close to the esophagus were enrolled.

The study was approved by the ethics review boards of our institution, and all patients were provided with signed informed consent prior to enrolment.

### 2.2. Treatment

Chemotherapy: The chemotherapy regimen consisted of etoposide (100 mg/m^2^ on days 1–3) and cisplatin (75 mg/m^2^ on day 1) administered once every 3 weeks for a total of 4 to 6 cycles. 

Based on a phase III trial demonstrating comparable efficacy between TRT initiated with the first or third cycle of chemotherapy [18], radiotherapy TRT was administered concurrently with the second or third cycle of chemotherapy. Patients underwent contrast-enhanced, four-dimensional, computed tomography (CT) simulation with a 3 mm slice thickness. The internal target volume (ITV) was defined as the post-chemotherapy residual primary tumour, including the internal margin for its respiratory motion. Positive lymph nodes were defined either with a short-axis diameter of at least 1 cm on the CT scan, or with an F-18 fluoro-2-deoxyglucose standard uptake value ≥2.5 on positron emission tomography/CT at initial staging, or with positive tumour cell sampling from lymph nodes. The CTV was created by expanding the ITV by 5 mm and containing positive prechemotherapy lymph node stations by referring to the International Association for the Study of Lung Cancer 2009 proposed lymph node map for lung cancer [19]. The planning target volume (PTV) was generated by a 5 mm expansion of the CTV. The prescription dose was 45 Gy in 3 Gy QD. For all patients, IMRT, including volumetric-modulated arc therapy (VMAT) treatment planning was performed (Pinnacle, Philips Medical Systems (Cleveland), Fitchburg, WI, USA). Treatment plans used 6-MV photons and typically no more than 7 beams (IMRT) or 4 arcs (VMAT) to minimize the low-dose exposure to the lungs. If VMAT was used, we used a virtual block method to spare normal lung tissue, especially for reductions in V5/V10 for the contralateral lung [20]. All of the important OARs were used for optimization with hard constraints, such as lung, heart, cord, and esophagus. In order to provide adequate dose coverage to the target, the maximum allowed dose to the esophagus was slightly higher than the PTV-prescribed dose while the esophagus overlapped within the target volume. The ITV was requested to receive 100% coverage of the prescribed dose. Additionally, 95% of the PTV was required to receive 100% of the prescribed dose.

Image-guided TRT with cone beam CT (CBCT) was delivered by 6 MV photon beams from linear accelerators. The mean dose to the lungs (MLD) should optimally be ≤15 Gy; thus, the lung volume minus ITV receiving more than 20 Gy (V20) was limited to less than 25%. The heart volumes receiving more than 30 Gy (V30) were limited to ≤40%; however, they were preferably lower. The maximum dose to the spinal cord was 40 Gy. The mean and maximal doses received by the esophagus were limited to less than 34 Gy and 50 Gy, respectively. A standard (non-SEAS) RT plan was created for each patient as a self-control plan.

### 2.3. Esophagus-Sparing Technique

**Figure 1:**
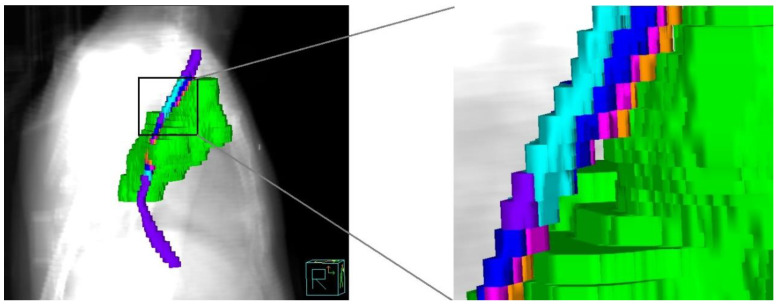
Segmental method of esophagus according to the isotropic distances in 3D directions to the planning target volume (PTV) geometry. PTV (green), involved esophagus (IE, white), abutting esophagus1 (AE1, orange), AE2 (light purple), AE3 (shaded blue), AE4 (blue), and AE5 (purple).

This study used a segmental abutting esophagus-sparing (SAES) technique for the whole esophagus. The entire esophagus was defined as extending from the inferior border of the cricoid cartilage to the gastroesophageal junction. The external surface of the entire esophagus was contoured on each axial slice of the CT images. In the SAES technique, the whole esophagus was divided into the involved esophagus (IE) and the abutting esophagus (AE), including six segmental contours: (1) IE: the esophagus involved in the planning target volume (PTV); (2) AE1: the esophagus outside the PTV at a distance of 0 to 3 mm away from the edge of the PTV; (3) AE2: the esophagus outside the PTV at a distance of 3 to 5 mm away from the edge of the PTV; (4) AE3: the esophagus outside the PTV at a distance of 5 mm to 1 cm away from the edge of the PTV; (5) AE4: the esophagus outside the PTV at a distance of 1 to 2 cm away from the edge of the PTV; and (6) AE5: the esophagus outside the PTV at a distance of 2 cm away from the edge of the PTV. All the distances of the segmental contours above were isotropic in 3D directions based on the PTV geometry (Figure 1). 

**Figure 2:**
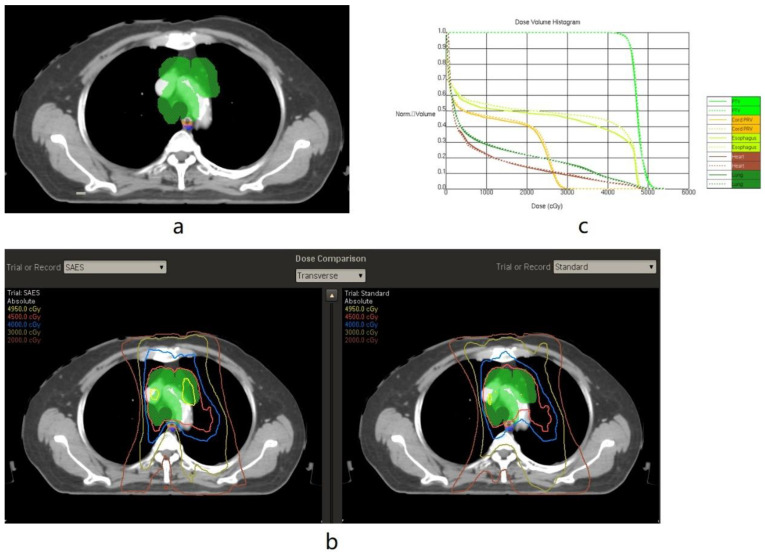
Illustrating the segmental abutting esophagus-sparing (SAES) technique applied in left-side tumour (Axial CT Images). (**a**): Examples of the planning target volume (green), involved esophagus (IE, white), abutting esophagus1 (AE1, orange), AE2 (light purple), and AE3 (shaded blue). (**b**): Examples of isodose line distributions showing effective AE-sparing sparing in the SAES plan compared to the standard (non-SAES) plan. In the SAES plan, isodose lines of 4000 and 4500 cGy were set to avoid AE1–3, and an isodose line of 3000 cGy was set to avoid AE3. (**c**) Examples of the dose-volume histogram (DVH) showing a reduced volume of the esophagus receiving 25–45 Gy in the SAES plan (solid line) compared with the standard plan (dotted line).

**Figure 3:**
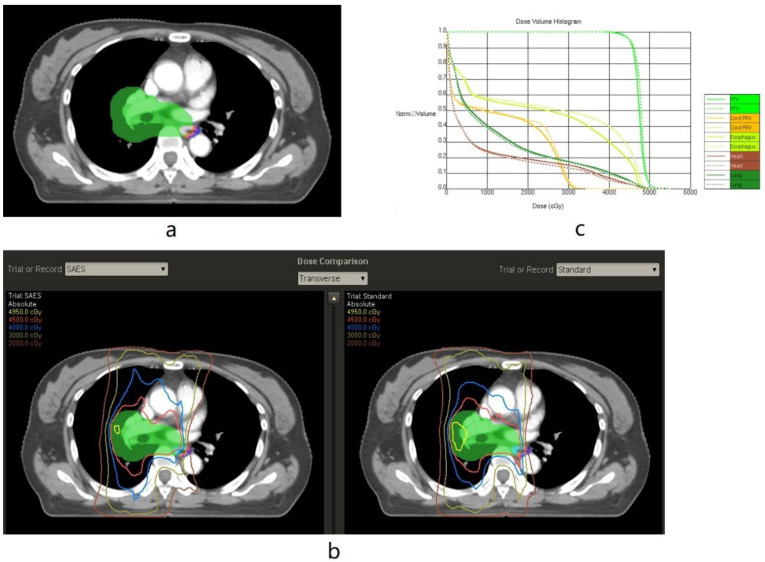
Illustrating the segmental abutting esophagus-sparing technique applied in right-side tumour (Axial CT Images). (**a**): Examples of planning target volume (green), involved esophagus (IE, white), abutting esophagus1 (AE1, orange), AE2 (light purple), and AE3 (shaded blue). In the SAES plan, an isodose line of 4000 cGy was set to avoid AE1–3, and an isodose line of 3000 cGy was set to avoid AE3. (**b**): Examples of isodose line distributions showing effective AE-sparing sparing in the SAES plan compared to the standard (non-SAES) plan. (**c**): Examples of dose-volume histogram (DVH) showing reduced volume of the esophagus receiving 30–45 Gy in the SAES plan (solid line) compared with the standard plan (dotted line).

The limitation of the maximum and mean doses received by the esophagus were the same as the standard RT plan (D mean <34 Gy and Dmax <50 Gy). However, the segmental contours’ maximum doses were constrained to guide a rapid dose fall-off gradient beyond the target volume, close to the esophagus. All of the segmental contours focused on reducing both the maximum dose and mean dose of the entire esophagus. Each segmental contour (AE 1–5) was given a lower maximum dose, usually 50% to 90% of the prescription dose, to create as sharp a dose gradient as possible. The dose constraints used for the segmental structures are listed in Supplementary. The target volume of the patient was labelled off and randomly assigned to one of two physicists to design the SAES treatment plan. Non-SAES plans were derived using a traditional IMRT/VMAT approach without using the SAES technique in Pinnacle by the other physicist (Figure 2 and Figure 3). Dose-volume histogram (DVH) parameters achieved with SAES versus non-SAES for each patient were compared. The SAES plan was delivered. 

### 2.4. Toxicity Evaluation and Follow-Up

Acute toxicity was graded according to the National Cancer Institute Common Terminology Criteria for Adverse Events (CTCAE 5.0) for AE between the start of radiotherapy and up to the 3-month post-treatment follow-up visit [21]. Late esophageal (LE) toxicity was defined as those occurring more than 90 days from the start of the TRT [22]. The primary endpoint was the rate of grade ≥3 (G3+) acute esophagitis. The charts of all patients were reviewed, and the grade of esophagitis was scored in 2-year follow-up visit since the start of treatment.

### 2.5. Statistical Analysis

According to the data of ≥grade 3 acute esophagitis by 45 Gy in 1.5 Gy BID (18.6–32%) [2,4,23] and 42 Gy in 2.8 Gy QD (31%) [9], we hypothesised the SAES technique was able to reduce G3+ acute esophagitis from 20% to 5%, and at least 29 patients were needed to be enrolled, according to optimal two-stage designs for phase II clinical trials (α = 0.05, β = 0.2) (Supplementary Table S2) [24]. 

After the HYPO TRT was completed, follow-up was conducted regularly until death or loss of follow-up. Living patients were censored at the last visit. The local regional recurrence (LR) was defined as clinical and/or biopsy-proven recurrence within the primary tumour, bronchial stump, ipsilateral hilum, mediastinum, or supraclavicular, irrespective of distant metastasis. The distant metastasis (DM) was defined as any evidence of metastatic disease beyond the locoregional regions. The local regional recurrence-free survival (LRFS), distant metastasis-free survival (DMFS), progress-free survival (PFS), and overall survival (OS) rates were calculated from the date of the first day of TRT with Kaplan–Meire method.

Continuous variables were described as means (standard deviation or range) and compared with the Student’s *t*-test. Qualitative variables were described as frequencies and percentages and compared with the Fisher’s exact or χ^2^ test. The Clopper–Pearson Exact Confidence interval was used to estimate the 95% confidence interval. Plan differences were analyzed with the Wilcoxon Signed-Rank Test. Statistical analyses were performed using SPSS version 22.0 (SPSS Inc., Chicago, IL, USA). All *p* values were two-tailed, and *p* < 0.05 was considered statistically significant.

## 3. Results

### 3.1. Characteristics of Patients

From 1 May 2021 to 30 April 2022, 30 patients who had their esophagus ≤1 cm from the CTV were enrolled and received TRT using the SAES technique (Supplementary Figure S1). Table 1 summarises the demographic, tumour, and treatment characteristics of the patients. Our patient population was predominantly male (66.7% men vs. 33.3% women), with a median age of 62 years. A majority of patients presented with Stage N2–3 (90.0%) and T2–4 (76.7%), in which four patients had ultracentral-located primary tumours according to the RTOG definition [25]. At diagnosis, only one (3.3%) patient was evaluated as stage II, and all other patients were diagnosed with stage III disease. Two patients received radiotherapy concurrently with the second cycle of chemotherapy, and twenty-eight patients underwent the third cycle. All patients completed four cycles of planned chemotherapy, except for one patient who had a second concurrent cycle delayed because of Grade 3 liver function damage. One patient received 42 Gy for Grade 3 acute esophagitis.

### 3.2. Dosimetric Advantage of SAES Technique

With the SAES technique, all dosimetric parameters were significantly reduced compared with the non-SAES plans, including the whole esophagus and AE (Table 2). The maximal and mean dose of the esophagus (47.4 ± 1.9 Gy and 13.5 ± 5.8 Gy, respectively) and AE (42.9 ± 2.3 Gy and 8.6 ± 3.6 Gy, respectively) in the SAES plan is significantly lower than those (esophagus 48.0 ± 1.9 Gy and 14.7 ± 6.1 Gy, AE 45.1 ± 2.4 Gy and 9.8 ± 4.2 Gy, respectively) in the non-SAES plan. Similar results were achieved for the volumes that received 30–45 Gy of the esophagus and AE. Esophageal sparing did not compromise PTV coverage or the homogeneity index of the plan, except for the conformation index (*p* = 0.03). For all lungs, the volumes received were 20 Gy (17.9 ± 3.2% vs. 18.0 ± 3.1%, *p* = 0.46) and 30 Gy (13.2 ± 2.5% vs. 13.2 ± 2.6%, *p* = 0.32), and were similar between SAES and non-SAES plans. The mean dose (9.0 ± 1.6 Gy vs. 9.0 ± 1.5 Gy, *p* = 0.02) and V10 (24.52 ± 5.29% vs. 24.32 ± 5.13%, *p* = 0.03) of all lungs, and mean dose of coronary artery (17.27 ± 8.66 Gy vs. 16.33 ± 7.08 Gy, *p* < 0.01) were slightly higher in the SAES plan compared with the non-SAES plans, but all the differences were quite low (≤1 Gy and <1%). There were no differences in the main dosimetric parameters for the cord and heart between SAES and non-SAES plans. The planning time was similar for SAES and between SAES and non-SAES plans radiotherapy (106.00 ± 28.15 min vs. 107.00 ± 23.35 min, *p* = 0.908).

### 3.3. Response to Treatment and Toxic Events

The response to chemoradiotherapy was assessed one month after the completion of the fourth cycle of chemotherapy. A complete response was found in eleven (36.7%) patients, partial response in eighteen (60.0%) patients, and stable response in one (3.3%) patient, according to RECIST V1.0. After the follow-up of more than 7 months (range, 7.0–18.1 months) for all patients, only one patient (3.3%, 95%CI 0.1%–17.2%) experienced grade 3 acute esophagitis and no grade 4–5 acute esophagitis occurred (Table 3). For late toxicities, one patient suffered sustained grade 1 late esophagitis and all others displayed no symptoms of esophagitis. The rate of radiation pneumonitis was very low, with one grade 3 event and no grade 4–5 events. Twelve (40.0%) patients had G3+ hematologic toxic events, including two patients with febrile neutropenia.

Thirty-Two patients in the experimental arm of NCT 02688036 from 1 Nov 2020 to 30 April 2021 were identified as the historical control group. The baseline characteristics are presented in Supplementary Table S3. With a median follow-up of 13.3 months (6.2–23.2 months), four patients developed acute G3 esophagitis (12.5% [95% CI 4.0–24.6%]), which caused three patients to fail to receive the full planned doses (one patient with 42 Gy, two patients with 39 Gy). Eight patients suffer late G1 esophagitis and four patients suffered G1 esophageal constriction (Supplementary Table S3). No G4-5 events occurred. These data demonstrated a high risk of esophagus-related toxicity when treated by non-SEAS HYPO TRT.

### 3.4. Outcomes

With a median follow-up of 12.5 months (range, 7.0–18.1 months), the 1-year OS, LRFS, DMFS, and PFS were 96.4%, 88.7%, 78.4%, and 64.3%, respectively. No patient developed local recurrence in the abutting esophagus-sparing region.

## 4. Discussion

To our knowledge, this is the first study to evaluate the feasibility and efficiency of the segmental abutting esophageal-sparing technique in patients with LS-SCLC treated with HYPO TRT. We divided the esophagus into five segments based on the location adjacent to the target volume. The doses delivered to the five segments of the esophagus were significantly reduced, which was successfully translated to clinical benefit with only 3.3% G3+ acute esophagitis and no G2+ late esophagitis. Compared with the historical control group, TRT with the SEAS technique truly reduced severe acute esophagitis and ensured more patients received full doses, which would have a positive impact on the prognosis.

The Intergroup (INT) 0096 trial reported a 5-year survival rate of 26% and a median OS of 23 months with 45 Gy in 1.5 Gy BID, establishing this regimen as an accepted standard of care for LS-SCLC [2,26]; although, the timing, dose, and fractionation are still under investigation. When the BID fraction is used, there should be at least a 6 h interfraction interval for the repair of normal tissue, which makes it logistically challenging for many patients and RT centers. In contrast to the HYPER schedule, HYPO TRT using >2.1 Gy per fraction is also practiced in certain parts of the world, with a common regimen being 40 Gy in 15 fractions QD [27]. Two prospective phase II trials implied 42 Gy in 15 fractions QD which is close to the current HYPO schedule and achieved relatively lower median OS (18–21 months) than those of the control group (45 Gy BID) and INT 0096 trial (*p* = 0.61) [9,13]. Another phase II trial and one retrospective study investigated HYPO schedules with a lower single dose but higher total dose (65 Gy in 26 fractions QD and 55 Gy in 22 fractions QD) [14,28], which resulted in a median OS of 27.2–39.3 months, non-inferior to the control groups (45 Gy BID) in each study. For early and late adverse effects, there are no differences between the HYPO group and HYPER group in the studies mentioned above; however, the rate of G3+ acute esophagitis for the HYPO group was still high, ranging from 13% to 31% in prospective trials [9,13,14].

Based on these results, the NCCN (Version 2.2022) panel recommends HYPO regimen as an alternative to the 45 Gy BID regimen; however, they did not clarify the optimal dose and fractions. The NCT 02688036 trial chose 45 Gy in 3 Gy QD, balancing dose escalation and the risk of toxicity in the acute and late term. This HYPO regimen was used in a few patients and did not increase severe toxicities compared with CF TRT [29]. In order to reduce esophagitis while exploring the effectiveness of 45 Gy in 15 fractions QD, the current study tested the SAES technique in 30 consecutive patients. Our results showed that only one patient (3.3%) had Grade ≥3 acute esophagitis, which was the lowest rate compared to all previous prospective trials and retrospective studies [9,10,11,12,13,14]. Furthermore, SAES did not increase planning time when compared to the standard plan.

These findings are of additional importance as they may be helpful in reducing the risk of esophageal toxicity without compromising the dose delivered to the tumour. For patients with a CTV overlapping or close to the esophagus, reducing the absolute high dose of the esophagus may compromise the target volume coverage by the prescription dose. Thus, we divided the esophagus into IE and AE, and further efforts were made to reduce the dose of AE. As a result, the doses to the whole esophagus and AE were significantly reduced by the SAES technique, which also provided an adequate dose covering the PTV compared with the standard plan. The dosimetric advantages were transformed into obvious clinical benefits, with only 3.3% G3+ esophagitis cases. Additionally, the dose to other normal organs was not elevated in the SAES technique plan compared with the standard plan, except for the mean lung dose. However, the difference in mean lung dose was very small, and the highest parameters were also low (≤13 Gy), which satisfied the dose limits of the whole plan. Therefore, the SAES technique did not increase other general toxicities, particularly pneumonitis. Our data showed the rates of radiation pneumonitis and haematological toxicities were comparable with historical publications [9,14,30].

One major limitation of the current study is the small sample size and single-center design; thus, the generalization of our findings may be weakened. However, the current study is unique in that the study cohort was derived from prospective phase III trials, and was the largest study compared with previous similar studies in lung cancer, which used totally different methods to sparring the esophagus [15,16]. The SAES treatment plans were randomly assigned to two physicists, who verified the feasibility and operability of this technique. The second major limitation is that the SEAS technique is equipment-depended, which requires CT simulators, a linear accelerator, and a treatment planning system for IMRT. Currently, the IMRT is available in over 70% of radiation centers in China and most radiation centers in western countries [31]. Theoretically, SEAS could be implied in these centers. The third limitation is that as the mean dose to the coronary artery was slightly increased, cardiac side events need to be noticed and close follow-ups over a long time need to be performed. Further investigations in other institutions and in a large number of patients are warranted, particularly for those with ultracentral tumours.

## 5. Conclusions

In conclusion, SAES radiotherapy has significant dosimetric advantages compared with standard radiotherapy, which could successfully translate into clinical benefits for LS-SCLC patients treated with HYPO daily fractions with concurrent chemotherapy. This method may help to achieve better tolerance of HYPO schedule and even better local control with dose escalation in the future.

## Figures and Tables

**Table 1:** Baseline data of patients.

Characteristic	n (%)
Age (median and range, years)	62 (46–73)
Gender	
Male	20 (66.7)
Female	10 (33.3)
KPS	
100	6 (20.0)
90	18 (60.0)
80	6 (20.0)
Smoking History	
Yes	20 (66.7)
No	10 (33.3)
Site	
Left lung	15 (50.0)
Right Lung	15 (50.0)
T stage	
T1	7 (23.3)
T2	11 (36.7)
T3	4 (13.3)
T4 *	8 (26.7)
N stage	
N1	3 (10.0)
N2	20 (66.7)
N3	7 (23.3)
Clinical Stage	
II	1 (3.3)
IIIa	15 (50.0)
IIIb	11 (36.7)
IIIc	3 (10.0)
ITV volume (median and range, cm^3^)	40.0 (7.6–116.9)
CTV volume (median and range, cm^3^)	148.6 (44.5–233.3)
PTV volume (median and range, cm^3^)	291.6 (118.1–448.3)
Radiotherapy dose	
42 Gy/3 Gy daily	1 (3.3%)
45 Gy/3 Gy daily	29 (96.7%)

*, Four patients had ultracentral tumours invaded mediastitum; CTV, Clinical target volume; ITV, Internal tumour volume; KPS, Karnofsky performance score; PTV, Planning target volume.

**Table 2:** Dose–volume histogram parameters between segmental free esophagus-sparing technique and standard radiotherapy.

		Segmental Abut Esophagus-Sparing Radiotherapy	Standard Radiotherapy	*p*-Value
		Mean ± SD	Mean ± SD	
PTV	D_mean_ (Gy)	47.64 ± 0.37	47.71 ± 0.40	0.23
	D_2%_ (Gy)	50.76 ± 1.05	50.83 ± 1.35	0.89
	D_98%_ (Gy)	44.04 ± 0.36	44.03 ± 0.38	0.99
	CI	1.34 ± 0.12	1.32 ± 0.11	0.03
	HI	0.14 ± 0.02	0.14 ± 0.03	0.72
MU		1120.66 ± 208.68	1082.42 ± 190.06	<0.001
Esophagus	D_max_ (Gy)	47.38 ± 1.93	47.96 ± 1.85	<0.001
	D_mean_ (Gy)	13.52 ± 5.76	14.66 ± 6.11	<0.001
	V_45_ (%)	9.8321 ± 9.04	11.87 ± 10.85	<0.001
	V_40_ (%)	14.48 ± 11.48	18.12 ± 13.76	<0.001
	V_35_ (%)	17.99 ± 13.18	22.34 ± 14.80	<0.001
	V_30_ (%)	21.76 ± 14.26	25.53 ± 15.21	<0.001
	V_25_ (%)	25.93 ± 15.3	28.45 ± 15.42	<0.001
	V_20_ (%)	29.26 ± 15.57	31.32 ± 15.42	<0.001
	V_10_ (%)	37.56 ± 14.49	39.09 ± 14.41	<0.001
AE	D_max_ (Gy)	42.91 ± 2.29	45.06 ± 2.39	<0.001
	D_mean_ (Gy)	8.55 ± 3.64	9.81 ± 4.24	<0.001
	V_45_ (cm^3^)	0.02 ± 0.04	0.30 ± 0.63	<0.001
	V_40_ (cm^3^)	0.51 ± 0.54	1.69 ± 1.64	<0.001
	V_35_ (cm^3^)	1.59 ± 1.52	3.04 ± 2.41	<0.001
	V_30_ (cm^3^)	2.88 ± 2.23	4.12 ± 2.79	<0.001
	V_25_ (cm^3^)	4.25 ± 2.88	5.09 ± 3.05	<0.001
	V_20_ (cm^3^)	5.38 ± 3.18	6.09 ± 3.20	<0.001
	V_10_ (cm^3^)	8.28 ± 3.57	8.82 ± 3.58	<0.001
AE1	D_max_ (Gy)	41.18 ± 2.58	44.12 ± 2.79	<0.001
AE2	D_max_ (Gy)	34.24 ± 3.66	40.2 ± 4.67	<0.001
AE3	D_max_ (Gy)	29.04 ± 5.11	36 ± 7.46	<0.001
AE4	D_max_ (Gy)	18.62 ± 7.49	24.22 ± 10.14	<0.001
AE5	D_max_ (Gy)	4.96 ± 2.59	5.63 ± 3.69	0.06
Lung	V_30_ (%)	13.15 ± 2.49	13.20 ± 2.60	0.32
	V_20_ (%)	17.91 ± 3.22	17.98 ± 3.10	0.46
	V_10_ (%)	24.52 ± 5.29	24.32 ± 5.13	0.03
	V_5_ (%)	31.84 ± 7.27	31.67 ± 7.25	0.06
	D_mean_ (Gy)	9.03 ± 1.53	8.98 ± 1.58	0.02
Cord	D_max_ (Gy)	27.89 ± 0.96	28.02 ± 1.07	0.24
Cord PRV	D_max_ (Gy)	30.55 ± 1.49	30.80 ± 1.38	0.24
Heart	V_40_ (%)	4.20 ± 3.68	3.90 ± 3.46	0.02
	V_30_ (%)	10.54 ± 8.14	10.50 ± 8.52	0.82
	D_mean_ (Gy)	8.43 ± 5.35	8.40 ± 5.42	0.58
Coronary artery	D_mean_ (Gy)	17.27 ± 8.66	16.33 ± 7.08	<0.01
	D_max_ (Gy)	39.11 ± 5.86	38.85 ± 4.34	0.19
Planning generation and optimization	Time (min)	106.00 ± 28.15	107.00 ± 23.35	0.91

AE, Abut esophagus (the esophagus outside the PTV); CI, Conformation Index; D_max_, Maximum dose which is defined as the dose received by 0.03 cc; D_mean_, Mean dose; D_x%_, Lowest dose received in any x% volume; HI, Homogeneity Index; ITV, Internal tumour volume; MU, Monitor units over all treatment beams; PTV, Planning target volume; PRV, The planning organ-at-risk volume; SD, Standard deviation; V_X_, Volume received x Gy.

**Table 3:** Acute radiation esophagitis and other toxic events.

	Esophagitis n (%) [95% CI]	Radiation Pneumonitis n (%) [95% CI]	Neutropenia n (%)	Lymphopenia n (%)	Thrombocytopenia n (%)	Anemia n (%)	Nausea n (%)
Grade 0–1	23 (76.7) [57.7–90.1]	27 (90.0) [73.5–97.9]	17 (56.7)	7 (23.3)	22 (73.3)	28 (93.3)	23 (76.6)
Grade 2	6 (20.0) [7.7–38.7]	2 (6.7) [0.8–22.1]	7 (23.3)	17 (56.7)	6 (20.0)	2 (6.7)	4 (13.3)
Grade 3	1 (3.3) [0.1–17.2]	1 (3.3) [0.1–17.2]	4 (13.3)	6 (20.0)	2 (6.7)	0 (0.0)	1 (3.3)
Grade 4	0 (0.0) [0–11.6]	0 (0.0) [0–11.6]	2 (6.6)	0 (0.0)	0 (0.0)	0 (0.0)	0 (0.0)

CI: Confidence interval. No Grade 5 events happened.

## Data Availability

Research data are stored in an institutional repository and will be shared upon request to the corresponding author.

## Supplementary Materials

Please use standard format: The following supporting information can be downloaded at: https://www.mdpi.com/article/10.3390/cancers15051487/s1, Table S1. Inclusion and exclusion criteria of NCT 02688036; Table S2. Sample size by optimal two-stage designs for phase II clinical trials; Table S3. Baseline characteristics and acute esophagitis of pre-SEAS cohort; Figure S1. Flow diagrams of included patients..

## References

[1] Rudin C.M., Brambilla E., Faivre-Finn C., Sage J. (2021). Small-cell lung cancer. Nat. Rev. Dis. Prim..

[2] Turrisi A.T., Kim K., Blum R., Sause W.T., Livingston R.B., Komaki R., Wagner H., Aisner S., Johnson D.H. (1999). Twice-daily compared with once-daily thoracic radiotherapy in limited small-cell lung cancer treated concurrently with cisplatin and etoposide. N. Engl. J. Med..

[3] Früh M., De Ruysscher D., Popat S., Crinò L., Peters S., Felip E. (2013). Small-cell lung cancer (SCLC): ESMO Clinical Practice Guidelines for diagnosis, treatment and follow-up. Ann. Oncol..

[4] Faivre-Finn C., Snee M., Ashcroft L., Appel W., Barlesi F., Bhatnagar A., Bezjak A., Cardenal F., Fournel P., Harden S. (2017). Concurrent once-daily versus twice-daily chemoradiotherapy in patients with limited-stage small-cell lung cancer (CONVERT): An open-label, phase 3, randomised, superiority trial. Lancet. Oncol..

[5] Pijls-Johannesma M., De Ruysscher D., Vansteenkiste J., Kester A., Rutten I., Lambin P. (2007). Timing of chest radiotherapy in patients with limited stage small cell lung cancer: A systematic review and meta-analysis of randomised controlled trials. Cancer Treat. Rev..

[6] De Ruysscher D., Pijls-Johannesma M., Bentzen S.M., Minken A., Wanders R., Lutgens L., Hochstenbag M., Boersma L., Wouters B., Lammering G. (2006). Time between the first day of chemotherapy and the last day of chest radiation is the most important predictor of survival in limited-disease small-cell lung cancer. J. Clin. Oncol..

[7] Kepka L., Danilova V., Saghatelyan T., Bajcsay A., Utehina O., Stojanovic S., Yalman D., Demiral A., Bondaruk O., Kuddu M. (2007). Resources and management strategies for the use of radiotherapy in the treatment of lung cancer in Central and Eastern European countries: Results of an International Atomic Energy Agency (IAEA) survey. Lung Cancer.

[8] Komaki R., Khalid N., Langer C.J., Kong F.M., Owen J.B., Crozier C.L., Wilson J.F., Wei X., Movsas B. (2013). Penetration of recommended procedures for lung cancer staging and management in the United States over 10 years: A quality research in radiation oncology survey. Int. J. Radiat. Oncol. Biol. Phys..

[9] Gronberg B.H., Halvorsen T.O., Flotten O., Brustugun O.T., Brunsvig P.F., Aasebo U., Bremnes R.M., Tollali T., Hornslien K., Aksnessaether B.Y. (2016). Randomized phase II trial comparing twice daily hyperfractionated with once daily hypofractionated thoracic radiotherapy in limited disease small cell lung cancer. Acta Oncol..

[10] Turgeon G.A., Souhami L., Kopek N., Hirsh V., Ofiara L., Faria S.L. (2017). Thoracic irradiation in 3weeks for limited-stage small cell lung cancer: Is twice a day fractionation really needed?. Cancer Radiother..

[11] Socha J., Guzowska A., Tyc-Szczepaniak D., Wierzchowski M., Sprawka A., Szczesna A., Kepka L. (2015). Accelerated hypofractionated thoracic radiotherapy in limited disease small cell lung cancer: Comparison with the results of conventionally fractionated radiotherapy. J. BUON.

[12] Sculier J.P., Lafitte J.J., Efremidis A., Florin M.C., Lecomte J., Berchier M.C., Richez M., Berghmans T., Scherpereel A., Meert A.P. (2008). A phase III randomised study of concomitant induction radiochemotherapy testing two modalities of radiosensitisation by cisplatin (standard versus daily) for limited small-cell lung cancer. Ann. Oncol..

[13] Bremnes R.M., Sundstrom S., Vilsvik J., Aasebo U. (2001). Multicenter phase II trial of paclitaxel, cisplatin, and etoposide with concurrent radiation for limited-stage small-cell lung cancer. J. Clin. Oncol..

[14] Qiu B., Li Q., Liu J., Huang Y., Pang Q., Zhu Z., Yang X., Wang B., Chen L., Fang J. (2021). Moderately Hypofractionated Once-Daily Compared With Twice-Daily Thoracic Radiation Therapy Concurrently With Etoposide and Cisplatin in Limited-Stage Small Cell Lung Cancer: A Multicenter, Phase II, Randomized Trial. Int. J. Radiat. Oncol. Biol. Phys..

[15] Al-Halabi H., Paetzold P., Sharp G.C., Olsen C., Willers H. (2015). A Contralateral Esophagus-Sparing Technique to Limit Severe Esophagitis Associated With Concurrent High-Dose Radiation and Chemotherapy in Patients With Thoracic Malignancies. Int. J. Radiat. Oncol. Biol. Phys..

[16] Kamran S.C., Yeap B.Y., Ulysse C.A., Cronin C., Bowes C.L., Durgin B., Gainor J.F., Khandekar M.J., Tansky J.Y., Keane F.K. (2021). Assessment of a Contralateral Esophagus-Sparing Technique in Locally Advanced Lung Cancer Treated With High-Dose Chemoradiation: A Phase 1 Nonrandomized Clinical Trial. JAMA Oncol..

[17] Shepherd F.A., Crowley J., Van Houtte P., Postmus P.E., Carney D., Chansky K., Shaikh Z., Goldstraw P. (2007). The International Association for the Study of Lung Cancer lung cancer staging project: Proposals regarding the clinical staging of small cell lung cancer in the forthcoming (seventh) edition of the tumor, node, metastasis classification for lung cancer. J. Thorac. Oncol..

[18] Sun J.M., Ahn Y.C., Choi E.K., Ahn M.J., Ahn J.S., Lee S.H., Lee D.H., Pyo H., Song S.Y., Jung S.H. (2013). Phase III trial of concurrent thoracic radiotherapy with either first- or third-cycle chemotherapy for limited-disease small-cell lung cancer. Ann. Oncol..

[19] Rusch V.W., Asamura H., Watanabe H., Giroux D.J., Rami-Porta R., Goldstraw P. (2009). The IASLC lung cancer staging project: A proposal for a new international lymph node map in the forthcoming seventh edition of the TNM classification for lung cancer. J. Thorac. Oncol..

[20] Chen J.-Y., Wang D.-Q., Zhang X.-D., Fu Q., Yan X.-N., Men K., Dai J.-R., Bi N. (2022). Sparing lung tissue with virtual block method in VMAT planning for locally advanced non-small cell lung cancer. Nucl. Sci. Tech..

[21] Cox J.D., Stetz J., Pajak T.F. (1995). Toxicity criteria of the Radiation Therapy Oncology Group (RTOG) and the European Organization for Research and Treatment of Cancer (EORTC). Int. J. Radiat. Oncol. Biol. Phys..

[22] Cannon D.M., Mehta M.P., Adkison J.B., Khuntia D., Traynor A.M., Tome W.A., Chappell R.J., Tolakanahalli R., Mohindra P., Bentzen S.M. (2013). Dose-limiting toxicity after hypofractionated dose-escalated radiotherapy in non-small-cell lung cancer. J. Clin. Oncol..

[23] Bogart J.A., Wang X., Masters G.A., Gao J., Komaki R., Gaspar L.E., Heymach J.V., Dobelbower M.C., Kuzma C., Stinchcombe T.E. (2021). Short Communication: Interim toxicity analysis for patients with limited stage small cell lung cancer (LSCLC) treated on CALGB 30610 (Alliance)/RTOG 0538. Lung Cancer.

[24] Simon R. (1989). Optimal two-stage designs for phase II clinical trials. Control. Clin. Trials.

[25] Timmerman R., McGarry R., Yiannoutsos C., Papiez L., Tudor K., DeLuca J., Ewing M., Abdulrahman R., DesRosiers C., Williams M. (2006). Excessive toxicity when treating central tumors in a phase II study of stereotactic body radiation therapy for medically inoperable early-stage lung cancer. J. Clin. Oncol..

[26] Schild S.E., Bonner J.A., Shanahan T.G., Brooks B.J., Marks R.S., Geyer S.M., Hillman S.L., Farr G.H., Tazelaar H.D., Krook J.E. (2004). Long-term results of a phase III trial comparing once-daily radiotherapy with twice-daily radiotherapy in limited-stage small-cell lung cancer. Int. J. Radiat. Oncol. Biol. Phys..

[27] Shahi J., Wright J.R., Gabos Z., Swaminath A. (2016). Management of small-cell lung cancer with radiotherapy-a pan-Canadian survey of radiation oncologists. Curr. Oncol..

[28] Zhang J., Fan M., Liu D., Zhao K.L., Wu K.L., Zhao W.X., Zhu Z.F., Fu X.L. (2017). Hypo- or conventionally fractionated radiotherapy combined with chemotherapy in patients with limited stage small cell lung cancer. Radiat. Oncol..

[29] Zayed S., Chen H., Ali E., Rodrigues G.B., Warner A., Palma D.A., Louie A.V. (2020). Is There a Role for Hypofractionated Thoracic Radiation Therapy in Limited-Stage Small Cell Lung Cancer? A Propensity Score Matched Analysis. Int. J. Radiat. Oncol. Biol. Phys..

[30] Gronberg B.H., Killingberg K.T., Flotten O., Brustugun O.T., Hornslien K., Madebo T., Langer S.W., Schytte T., Nyman J., Risum S. (2021). High-dose versus standard-dose twice-daily thoracic radiotherapy for patients with limited stage small-cell lung cancer: An open-label, randomised, phase 2 trial. Lancet Oncol..

[31] Zhang Y., Yi J.-L., Jiang W., Liu J.-P., Ma K.-K., Kang S.-G., Lang J.-Y., Wang J.-J., Ren H., Li B.-S. (2020). Survey on the Basic Information of Personnel and Facilities of Radiotherapy in Chinese Mainland in 2019. China Cancer.

