# Supplementary material for: Effect of Segmental Abutting Esophagus-Sparing Technique to Reduce Severe Esophagitis in Limited-Stage Small-Cell Lung Cancer Patients Treated with Concurrent Hypofractionated Thoracic Radiation and Chemotherapy
Source: Cancers (Basel). 2023 Feb 27;15(5):1487. doi: 10.3390/cancers15051487 (PMC10001206; doi:10.3390/cancers15051487)
Supplement: Supplementary file 1 [file cancers-15-01487-s001.zip › cancers-2206978-supplementary.pdf]

Supplementary Materials: Effect of Segmental Abutting Esophagus-Sparing Technique to Reduce Severe Esophagitis in Limited-Stage Small-Cell Lung Cancer Patients Treated with Concurrent Hypofractionated Thoracic Radiation and Chemotherapy

Jianyang Wang, Fei Han, Yuchao Ma, Yufan Yang, Yuqi Wu, Zimin Han, Xuejie Xie, Jianrong Dai, Nan Bi and Luhua Wang

Table S1. Inclusion and exclusion criteria of NCT 02688036.

Inclusion Criteria
• 18~70 years old, ECOG 0-2
• patients with histologically or cytologically proved small cell lung cancer
• Limited disease (LD), was characterized by tumors confined to one hemithorax. It may include ipsilateral hilar, both mediastinum and both supraclavicular nodes. Metastatic lymph nodes were defined as lymph nodes with short diameter of more than 1cm or FDG uptake in PET. The thickness of pleural effusion was less than 1cm unless malignant pleural effusion was cytologically proved. In a word, stage I-IIIB excluding the patients with lung metastases should be defined as LD according to AJCC cancer staging 7th edition.
• No progression after 2 cycles of chemotherapy with EP.
• No prior history of anti-tumor treatment.
• No severe internal diseases and no organ dysfunction
• Written informed consent provided and
**Exclusion Criteria**
• Malignant tumors of other sites. Non melanoma skin cancer and Cervical carcinoma in situ were not included if curable.
• Active heart disease or acute myocardial infarction happen in six months.
• Psychiatric history.
• Pregnant woman or woman need to breast feed or woman with positive chorionic gonadotrophin (HCG)
• Uncontrolled diabetes or hypertension
• Interstitial pneumonia or Active pulmonary fibrosis
• Acute bacterial or fungal infection
• Oral or intravenous use of steroids

The dose constraints used for the segmental structures in the esophagus-sparing included involved esophagus (IE) maximum dose of 110% of the prescription dose (IE<49.5Gy), abut esophagus (AE)1 maximum dose of 105% of the prescription dose (AE1<47.25Gy), AE2 maximum dose of 95% of the prescription dose (AE2<42.75Gy), AE3 maximum dose of 90% of the prescription dose (AE3<40.5Gy), AE4 maximum dose of 80% of the prescription dose (AE4<36Gy) and AE5 maximum dose of 50% of the prescription dose (AE5<22.5Gy), respectively. Efforts were made to limit the mean dose of all AE segments as low as possible. The segmental abut esophagus-sparing (SAES) technique was proceeded while avoiding excessive sacrifice of target area coverage. Acceptable plans were required to achieve: (1) 100% coverage of the internal target volume (ITV) by 100% of the prescribed dose, (2) 99% coverage of the clinical target volume (CTV) by 100% of the prescribed dose, (3) 95% coverage of the planning target volume (PTV) by 100% of the prescribed dose.

Sample size estimation (https://linus.nci.nih.gov/brb/samplesize//otsd.html)

- Study design: we hypothesized SAES technique was able to reduce Grade≥3 esophagitis from 20% to 5%. Then we get the sample size as following.
  α=0.05, β=0.2
- Response Probability (rate of grade 0-2 esophagitis) of standard radiotherapy (P0) → 0.80
- Response Probability (rate of grade 0-2 esophagitis) of SAES radiotherapy (P0)→ 0.95

Table S2. Sample size by optimal two-stage designs for phase II clinical trials

	**Optimum design**
First Stage Sample Size (n1)	9
Upper Limit For 1st Stage Rejection of SAES radiotherapy (r1)	7
Maximum Sample Size (n)	**29**
Upper Limit for 2nd Stage Rejection of SAES radiotherapy (r)	26
Expected Sample Size If Response Probability = P0	17.72
Probability of Early Termination at P0	0.56

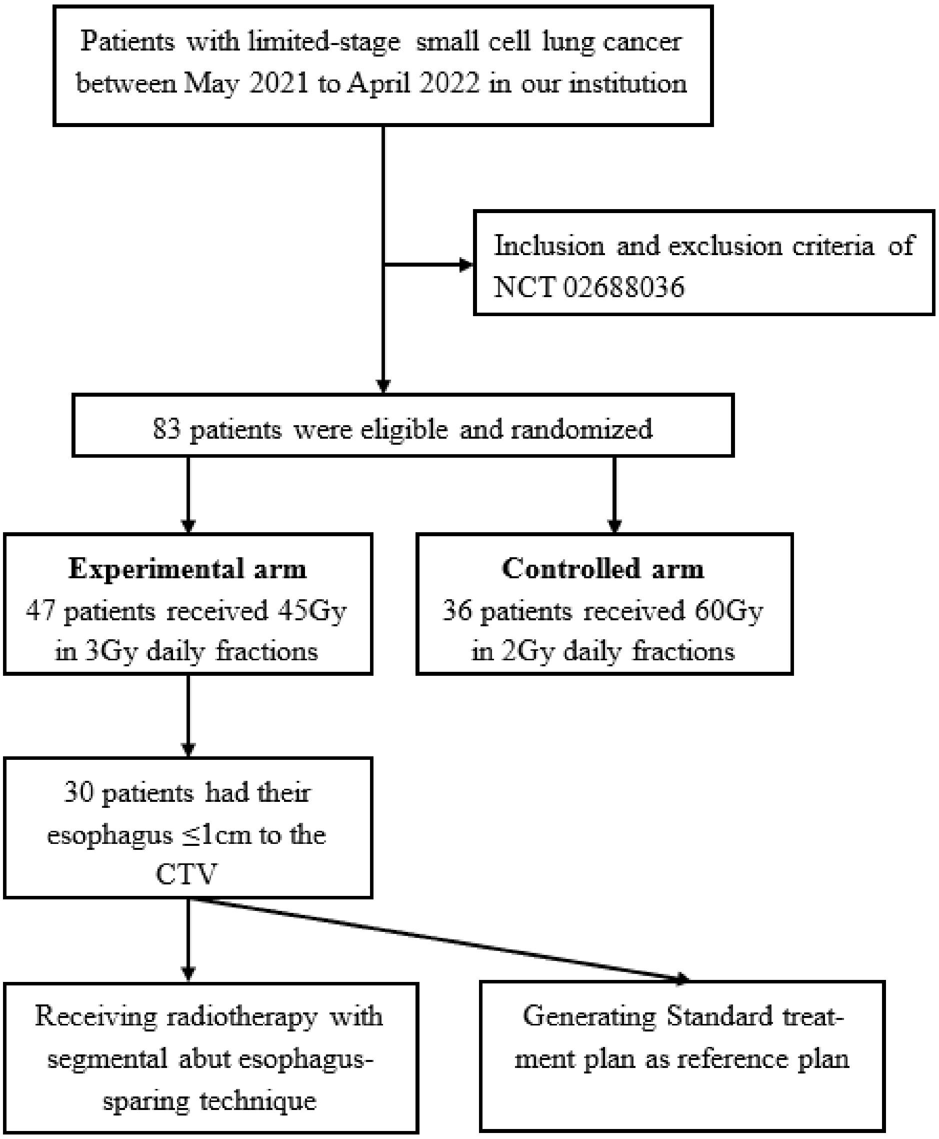

Figure S1. Flow diagrams of included patients.

Table S3. Baseline characteristics and acute esophagitis of pre-SEAS cohort

Characteristic	n (%)
Age (median and range, years)	61 (32-73)
Gender	
Male	25 (78.1)
Female	7 (21.9)
KPS	
100	7 (21.9)
90	24 (75.0)
80	1 (3.1)
Smoking History	
Yes	23 (71.9)
No	9 (28.1)
Site	
Left lung	16 (50.0)
Right Lung	16 (50.0)
T stage	
T1	5 (15.6)
T2	7 (21.9)
T3	12 (37.5)
T4*	8 (25.0)
N stage	
N1	11 (34.4
N2	16 (50.0
N3	5 (15.6)
Clinical Stage	
II	5 (15.6)
IIIa	10(31.3)
IIIb	13 (40.6)
IIIc	4 (12.5)
Radiotherapy dose	
24 Gy/3Gy daily	1 (3.1)
39 Gy/3Gy daily	2 (6.3)
42 Gy/3Gy daily	1 (3.1)
45 Gy/3Gy daily	28 (87.5)
Acute esophagitis	
Grade 0-1	18 (56.2) [95%CI 38.1-74.4]
Grade 2	10 (31.3) [95%CI 16.7-50.1]
Grade 3	4 (12.5) [95%CI 4.1-29.9]
Grade 4	0 (0.0) [95%CI 0-13.3]

KPS, Karnofsky performance score; No Grade 5 events happened.
